# Progress of antibody-based inhibitors of the HGF–cMET axis in cancer therapy

**DOI:** 10.1038/emm.2017.17

**Published:** 2017-03-24

**Authors:** Ki-Hyun Kim, Hyori Kim

**Affiliations:** 1 Cancer Research Institute, College of Medicine, Seoul National University, Jongno-gu, Seoul, Republic of Korea; 2 Department of Biochemistry and Molecular Biology, College of Medicine, Seoul National University, Jongno-gu, Seoul, Republic of Korea; 3 Institute for Life Sciences, ASAN Medical Center, Songpa-gu, Seoul, Republic of Korea

## Abstract

Dysregulated receptor tyrosine kinase signaling in human cancer cells leads to tumor progression, invasion and metastasis. The receptor tyrosine kinase cMET is frequently overexpressed in cancer tissue, and activation of cMET signaling is related to drug resistance and the processes of carcinogenesis, invasion and metastasis. For that reason, cMET and its ligand, hepatocyte growth factor (HGF), are considered prime targets for the development of anticancer drugs. At least eight anti-cMET and four anti-HGF antibodies have been tested or are being tested in clinical trials. However, to date none of these HGF/cMET inhibitors have shown significant efficacy in clinical trials. Furthermore, no receptor tyrosine kinase inhibitors primarily targeting cMET have been approved. Given that neutralization of HGF or cMET does not cause significant adverse effects, inhibition of the HGF/cMET signaling pathway appears to be safe. In this review, we summarized the completed and ongoing clinical trials testing antibody- or protein-based anticancer drugs targeting cMET and HGF.

## Introduction

The receptor tyrosine kinase cMET and its only known ligand, hepatocyte growth factor (HGF), play crucial roles in cellular proliferation, survival, invasion, tissue development and organ regeneration. cMET is produced as a single-chain precursor and converted by post-translational modification to form a structure that is linked by disulfide bonds. Mature cMET consists of a 50-kDa extracellular α-chain and a 140-kDa transmembrane β-chain.^1^ HGF is secreted as an inactive precursor (pro-HGF) that is activated through cleavage by serine proteases. Consequently, the active ligand structure of HGF consists of an N-terminal domain and Kringle domains (K1–K4) in the α-chain and a serine protease-like domain in the β-chain.^2^ The N-terminal domain and the K1 mediate high-affinity binding to cMET, which appears to induce the formation of a secondary binding site within the HGF β-chain. With this subsequent binding to cMET, HGF forms a strong complex that can induce signal transduction.^3^ The binding of active HGF to cMET leads to receptor multimerization and internalization, multiple phosphorylation of tyrosine residues in the intracellular kinase domain and subsequent activation of numerous signaling cascades related to cancer progression, invasion and metastasis.^4^

Despite tight regulation of HGF-induced cMET activation, dysregulated HGF–cMET signaling is observed in multiple malignant neoplasms.^5^ Aberrant cMET activation can occur through HGF-independent mechanisms such as *MET* mutations, gene amplification and transcriptional upregulation.^6^ cMET is overexpressed in a number of solid tumors, including brain cancer, breast cancer, colorectal cancer, gastric cancer, head and neck cancer, lung cancer, liver cancer, skin cancer, prostate cancer and soft tissue cancers.^4, 7, 8^ cMET can also be activated by interaction with epidermal growth factor receptor (EGFR). Given the resistance to EGFR tyrosine kinase inhibitors in cMET-expressing lung cancer and the synergistic effect of cMET and EGFR inhibitors, dual targeting of EGFR and cMET is a promising therapeutic strategy.^9, 10, 11^

Elevated tumor and plasma HGF levels are also observed in patients with certain types of cancer such as invasive breast carcinoma, glioma, multiple myeloma and sarcomas.^12, 13, 14, 15^ Several *in vivo* studies have shown that activation of the HGF–cMET signaling pathway triggers cancer invasion and metastasis.^16, 17, 18, 19^ Thus, multiple therapeutic agents that target the HGF–cMET pathway in various cancers are under development. For example, several monoclonal antibodies (mAbs) inhibit the HGF–cMET axis by blocking the binding of HGF to cMET or by targeting cMET on the cell surface. The safety profiles of these agents are better than those of small chemicals because mAbs have excellent target specificity and predictable pharmacological properties. Adverse effects and dose-limiting toxicities have been reported for small-molecule inhibitors, but few dose-limiting toxicities have been reported for mAbs.^20^ Numerous mAbs targeting the HGF–cMET signaling pathway with different mechanisms of action have been tested recently in patients with solid tumors (Table 1). This review summarizes the features of these antibodies or related proteins targeting the HGF–cMET axis and recent clinical findings.

## Anti-cMET antibodies

### Onartuzumab (MetMAb)

Many of the cMET antagonists developed so far are bivalent (two-armed) antibodies, which induce receptor crosslinking and downstream signaling after binding to cMET. However, onartuzumab is a monovalent antibody generated with Fab fragments with murine variable heavy and light domains fused to human IgG1 constant domains.^21^ Onartuzumab blocks the high-affinity binding of the HGF α-chain to cMET but not the binding of the HGF β-chain.^22^ Several preclinical studies have investigated the treatment efficacy of onartuzumab. In an intracranial xenograft mouse model of human glioblastoma, onartuzumab treatment decreased tumor size by 98.7%.^23^ In an orthotopic xenograft mouse model of pancreatic cancer, onartuzumab abolished tumor growth, decreased cMET phosphorylation and improved survival.^24^ A phase I study evaluated the safety and efficacy of single-agent onartuzumab, as well as combination treatment with onartuzumab plus bevacizumab in patients with locally advanced and metastatic solid tumors. A maximum tolerated dose was not reached, and the half-life was 11 days. In addition, no adverse pharmacokinetic interactions with bevacizumab were observed.^25^ In a phase II clinical study of onartuzumab in combination with erlotinib for advanced non-small cell lung cancer (NSCLC), the addition of onartuzumab to erlotinib significantly improved progression-free survival and overall survival (OS) in patients with positive immunohistochemical staining for cMET.^26, 27^ In contrast, a phase II study of patients with predominantly cMET-negative metastatic triple-negative breast cancer found that combining onartuzumab with paclitaxel with or without bevacizumab did not provide clinical benefit.^28^ In addition, a randomized, double-blind, phase III study of onartuzumab plus erlotinib in patients with cMET-positive advanced NSCLC was stopped because of lack of clinical efficacy. In this study, median OS was 6.8 months for the onartuzumab plus erlotinib arm and 9.1 months for the erlotinib plus placebo arm (hazard ratio, 1.27; 95% confidence interval, 0.98–1.65; *P*=0.07).^29^ Further development of onartuzumab has been halted.

### Emibetuzumab (LY2875358)

Emibetuzumab is a bivalent humanized anti-cMET IgG4 mAb that blocks HGF binding to cMET. Binding of emibetuzumab to cMET promotes internalization and degradation of cMET, in contrast to onartuzumab, which does not induce cMET degradation. Emibetuzumab inhibited both HGF-dependent and -independent tumor growth in mouse xenograft models.^30^ A recent first-in-human phase I clinical study tested single-agent emibetuzumab in patients with solid tumors and emibetuzumab in combination with erlotinib in patients with NSCLC (NCT01287546).^31^ No dose-limiting toxicities and adverse events were observed, and half-life was ~11 days. Of the 23 patients receiving emibetuzumab monotherapy, one patient achieved a partial response (objective response rate=4.3%) and five patients (21.7%) achieved stable disease. Two of the 14 NSCLC patients receiving combination treatment achieved a partial response (objective response rate=14.3%) and four (28.6%) achieved stable disease. Phase II clinical studies of emibetuzumab in NSCLC patients with *EGFR* mutations (NCT01897480) and combination treatment with the anti-VEGFR2 mAb ramucirumab (NCT02082210) are ongoing.

### LY3164530

LY3164530 is a bispecific anti-EGFR/cMET antibody generated by fusing an anti-EGFR single-chain variable fragment (humanized cetuximab sequence) to the N-terminus of the emibetuzumab heavy chain. LY3164530 binds and internalizes cMET and EGFR without agonistic activity. In a NSCLC xenograft model, LY3164530 showed better antitumor efficacy than combination treatment with emibetuzumab and cetuximab.^32^ A phase I clinical study is ongoing (NCT02221882).

### JNJ-61186372

The bispecific EGFR/cMET antibody JNJ-61186372 is a heterodimeric IgG1 composed of two units targeting EGFR and cMET.^33^ The cMET-binding IgG1 molecule is generated with a K490R mutation in the CH3 domain and the EGFR-binding IgG1 molecule is generated with a F405L mutation in the CH3 domain. With these IgG1 molecules, heterodimeric IgG is produced using the controlled Fab-arm exchange method.^34^ JNJ-61186372 inhibits tumor cell growth by the downregulation of both EGFR and cMET in combination with enhanced antibody-dependent cell-mediated cytotoxicity.^33, 35, 36, 37^ Patients with NSCLC with *EGFR* mutations are being recruited for a phase I clinical study (NCT02609776).

### SAIT301

SAIT301 is a monoclonal humanized antibody that promotes Casitas B-lineage lymphoma (CBL)-independent and leucine-rich repeats and immunoglobulin-like domain-containing protein 1 (LRIG1)-mediated cMET degradation.^38^ SAIT301 inhibits the invasion and migration of nasopharyngeal cancer cells by downregulating EGR-1 expression.^39^ In cMET-positive gastric cancer cell lines treated with SAIT301 in combination with paclitaxel, cMET was downregulated and poly(ADP ribose) polymerase (PARP) cleavage was enhanced compared to paclitaxel monotherapy.^40^ A phase I clinical study is currently recruiting patients with cMET-positive solid tumors (NCT02296879).

### ABT-700 (h224G11)

ABT-700 (previously known as h224G11) is a humanized, bivalent monoclonal antibody that inhibits cMET dimerization and activation that is induced by HGF or by the high density of cMET on the cell surface (independent of ligand). ABT-700 inhibits tumor growth in mice bearing gastric and lung cancer xenografts with *MET* amplification.^41^ A phase I study of ABT-700 alone and in combination with docetaxel, 5-fluoruracil, folinic acid, irinotecan and cetuximab (FOLFIRI/cetuximab) or erlotinib is ongoing in patients with advanced solid tumors that may have *MET* amplification or cMET overexpression (NCT01472016).

### ARGX-111

ARGX-111 is an afucosylated IgG1 antibody characterized by improved tissue penetration and enhanced antibody-dependent cellular cytotoxicity.^42^ ARGX-111 inhibits ligand-dependent cMET activation and shows cytotoxic activity in both cMET-expressing human cancer cells and patient-derived chronic lymphocytic leukemia and acute myeloid leukemia cells. In an orthotopic mouse model, ARGX-111 decreased the number of circulating tumor cells and suppressed metastasis.^43^ This drug is currently under clinical evaluation in a phase I trial in patients with advanced cancers overexpressing cMET (NCT02055066).

### DN30

DN30 is a monovalent chimeric Fab that induces the proteolytic cleavage of cMET, which causes the release of the soluble receptor and rapid proteasomal degradation of the intracellular portion.^44^ DN30 Fab reduces both HGF-dependent and HGF-independent tumor cell growth *in vitro*^45^ and delays tumor growth in preclinical models of human gastric cancer, lung carcinoma, and glioblastoma. However, because of its small molecular size, DN30 Fab is rapidly eliminated by the kidney. PEGylation and gene delivery have been proposed to overcome this drawback.^46, 47^

## Anti-HGF antibodies

### Rilotumumab (AMG-102)

Rilotumumab is a fully human anti-HGF IgG2 antibody that binds to the HGF β-chain, thereby inhibiting HGF–cMET binding.^48^ It is the first HGF inhibitor to reach phase III development. Results of a phase I trial in patients with solid tumors showed a maximum tolerated dose of 20 mg kg^−1^ every 2 weeks and a mean half-life of 15.4 h. All adverse events (fatigue, constipation, anorexia and nausea/vomiting) were low-grade.^49^ A phase II study demonstrated that adding rilotumumab to epirubicin, cisplatin and capecitabine (ECX) chemotherapy extended OS and progression-free survival in MET-positive patients with gastric cancer or gastroesophageal junction adenocarcinoma.^50^ Based on the positive results in phase II study, two phase III clinical trials (RILOMET-1 and RILOMET-2) were initiated. The RILOMET-1 study was stopped early in November 2014 because of lack of efficacy. At the data cutoff point, 128 deaths were observed in the rilotumumab arm, and 107 deaths were observed in the placebo arm, and a significantly shorter median OS was observed in rilotumumab arm compared with the control group (median OS 9.6 months vs 11.5 months, hazard ratio: 1.37, *P*=0.016). No subgroup benefitted from rilotumumab treatment, including patients with higher *MET* expression.^51^

### Ficlatuzumab (AV-299)

Ficlatuzumab is a humanized anti-HGF antibody. In a phase I trial, ficlatuzumab monotherapy was well tolerated in patients with solid tumors receiving 2, 5 or 10 mg kg^−1^ or the maximum dose of 20 mg kg^−1^. The most commonly observed toxicities were peripheral edema, fatigue and nausea. In this study, ficlatuzumab was administered to 37 patients with solid tumors either as a single agent (24 patients) or in combination with a daily 150 mg dose of erlotinib (13 patients). The best overall response (monotherapy or combination therapy) was stable disease (four cycles or 8 weeks), which was observed in 44% of the evaluable patients. In all ficlatuzumab-treated patients, serum HGF levels were increased compared with baseline, likely due to the stabilization of HGF upon complex formation with ficlatuzumab.^52^ A phase Ib trial evaluated combination treatment with ficlatuzumab and gefitinib in 15 molecularly unselected Asian NSCLC patients. At the 20 mg kg^−1^ dose, five patients had a partial response and four had stable disease after 12 weeks of treatment.^53^ The half-life of single-agent ficlatuzumab was approximately 7–10 days.^54, 55^ A multicenter, open-label, randomized phase II study evaluated combination treatment with ficlatuzumab and gefitinib versus gefitinib alone in Asian patients with lung adenocarcinoma not selected for *EGFR* mutational status. In a subgroup of patients with *EGFR* mutations and low cMET expression, patients treated with ficlatuzumab combined with gefitinib showed an improved objective response rate (41% vs 22%) and median progression-free survival (11 vs 5.5 months). However, in the overall population no significant difference was observed in response rate (40 vs 43% in the gefitinib arm vs combination arm, respectively) or progression-free survival (4.7 months vs 5.6 months in the gefitinib arm vs combination arm, respectively).^54, 56^ Results of a phase II study in a pulmonary adenocarcinoma population enriched for activating *EGFR* mutations showed that combination treatment with ficlatuzumab and gefitinib did not improve clinical outcomes compared to gefitinib monotherapy. Patients who are classified as VeriStrat-poor may benefit from the addition of ficlatuzumab.^57^ However, a recent phase II study (NCT02318368) comparing first-line treatment with ficlatuzumab plus erlotinib versus erlotinib monotherapy in VeriStrat-poor patients with *EGFR*-mutated NSCLC was discontinued because of a high discontinuation rate in the combination treatment arm.

### HuL2G7 (TAK-701)

HuL2G7 is a humanized antibody shown to overcome gefitinib resistance in *EGFR*-mutated human NSCLC cell lines.^58^ A phase I study conducted in patients with advanced solid malignancies showed that HuL2G7 had no specific dose-limiting toxicities, but adverse events included cough, abdominal pain, constipation and fatigue.^59^ Further clinical trials have not been pursued.

### YYB-101

YYB-101 is a humanized rabbit anti-HGF antibody. The preclinical development of this drug is described by Kim *et al.* in this issue. YYB-101 binds to the HGF α-chain, where the first high-affinity binding with cMET takes place (unpublished data). This drug inhibits cMET activation and cell scattering *in vitro* and tumor growth in several xenograft mouse models.^60, 61^ A phase I clinical study in patients with advanced solid tumors is ongoing (NCT02499224).

## Antibody mimetic engineered protein

### MP0250

MP0250 is a designed ankyrin repeat protein that neutralizes both HGF and vascular endothelial growth factor. The ability to bind to human serum albumin extends the plasma half-life of MP0250 to 12 days.^62^ As monotherapy and in combination with bortezomib, MP0250 inhibits myeloma cell migration, invasion and bone destruction in an orthotopic mouse model of multiple myeloma.^63^ Phase I interim results showed that MP0250 monotherapy was well tolerated, and a maximum tolerated dose had not been reached. Stable disease for 10 months was observed in one patient with a head and neck tumor and for 8 months in a patient with cervical adenocarcinoma.^64^ Based on preclinical and phase I study results, a phase II study is planned to evaluate MP0250 combined with bortezomib and dexamethasone in patients with multiple myeloma who have failed standard therapies.

## Competitive analogs of HGF

### NK4

NK4 is a synthetic molecule that consists of the N-terminal domain and four Kringle domains of HGF and lacks 16 amino acids from the HGF C-terminus. This drug binds cMET without activating signal transduction and exerts anti-growth, anti-metastasis and anti-angiogenic actions in various cancer animal models.^19^ In preclinical studies, adenoviruses expressing the *NK4* gene have demonstrated antitumor effects in numerous types of tumors including mesothelioma and pancreatic cancer.^65, 66^ A phase I clinical study of gene therapy using the *NK4*-expressing adenoviral vector in patients with mesothelioma is ongoing.^67^

### Other HGF mimic molecules

Uncleavable pro-HGF, generated by introducing a point mutation at the cleavage site (Arg494Gln), inhibits HGF-mediated cMET activation *in vitro* and local or systemic expression of pro-HGF suppresses tumor growth and prevents metastatic dissemination in mice.^19^ Mutations near the pocket region of the HGF protein (D672N, V495G, V495A, G498I and G498V) inhibit cMET activation because the N-terminal active pocket present in the HGF β-chain is required to stabilize interactions with cMET. Mutated HGF proteins suppress cMET phosphorylation and cancer cell migration *in vitro.*^68^

## Conclusion

The HGF–cMET pathway is a promising target for cancer therapy. A number of antibodies and engineered-proteins targeting HGF or cMET have been shown to be safe in clinical trials. However, none have demonstrated a significant clinical benefit to patients, with the exception of ficlatuzumab, which was shown to provide benefit to patients classified as VeriStrat-poor in a retrospective analysis of a failed clinical trial. Thus, better biomarkers may be needed for patient selection, or different clinical indications considered for future trials.

## Figures and Tables

**Table 1:** Antibodies targeting the HGF–cMET axis in development

*Drug name*	*Developer*	*Format*	*Indication*	*Clinical trial status*	*Clinical trial identifier*
*Anti-HGF antibodies*					
YYB-101	Yooyoung Pharmaceutical/National OncoVenture	mAb	Solid tumors	Phase I, active	NCT02499224
Rilotumumab (AMG-102)	Amgen	mAb (IgG2)	Colorectal cancer, Renal cell carcinoma, Glioma, Gastric cancer, Esophageal cancer	Phase II, Phase III	NCT00770848 NCT00422019 NCT00427440 NCT02137343 NCT01697072 NCT02926638 NCT00719550
Ficlatuzumab (AV-299, SCH900105)	AVEO Pharmaceuticals, Inc./Biodesix Inc.	mAb (IgG1)	Non-small cell lung cancer	Phase II	NCT02318368 NCT01039948
HuL2G7 (TAK-701)	Galaxy Biotech, LLC/Millennium Pharmaceuticals, Inc.	mAb (IgG1)	Advanced solid tumors	Phase I	NCT00831896
					
*Anti-cMET antibodies*					
SAIT301	Young Suk Park/Samsung Medical Center	mAb	Solid tumors	Phase I, active	NCT02296879
ARGX-111	arGEN-X BVBA	mAb	c-MET-overexpressing cancer	Phase I, active	NCT02055066
Onartuzumab (MetMAb, RO5490258)	Genentech, Inc./Hoffmann-La Roche	mAb (IgG1)	Glioblastoma, Non-small cell lung cancer, Gastric cancer, Breast cancer, Colorectal cancer	Phase II, Phase III	NCT01632228 NCT01456325 NCT02031744 NCT01887886 NCT01662869 NCT02488330 NCT01186991 NCT01418222
Emibetuzumab (LY2875358, LA480)	Eli Lilly & Company	mAb (IgG4)	Advanced cancer, Gastric cancer, Non-small cell lung cancer	Phase II	NCT02082210 NCT01874938 NCT01900652 NCT01897480
ABT-700 (h244G11)	AbbVie	mAb	Advanced solid tumors	Phase I	NCT01472016
JNJ-61186372	Janssen Research & Development, LLC	bsAb (DuoBody)	Non-small cell lung cancer	Phase I, active	NCT02609776
DN30	Metheresis Translational Research S.A.	Fab		Investigational	
LY3164530	Eli Lilly & Company	bsAb	Neoplasms	Phase I	NCT02221882
					
*Antibody mimetic engineered protein*					
MP0250	Molecular Partners AG	Designed ankyrin repeat protein	Advanced solid tumors	Phase I/II, active	NCT02194426
					
*Competitive analog of HGF*					
Ad-NK4	Chiba University	Adenovirus gene therapy		Investigational	

Abbreviations: bsAb, bispecific antibody; Fab, antigen-binding fragment; HGF, hepatocyte growth factor; mAb, monoclonal antibody.
